# Supplementary material for: Environmental factors affecting honey bees (Apis cerana) and cabbage white butterflies (Pieris rapae) at urban farmlands
Source: PeerJ. 2023 Jul 26;11:e15725. doi: 10.7717/peerj.15725 (PMC10386823; doi:10.7717/peerj.15725)
Supplement: Supplemental Information 1 — The mean value, i.e., %Avg was calculated from 90 sites used for cabbage white butterfly analysis. Although “orchard” is unidentified fruit trees, it was the only fruit tree species other than banana (and papaya), which do not belong to the same family. Thus, it was considered for analysis. [file peerj-11-15725-s001.docx]

| **Family** | **%Avg** |  | **Family** | **%Avg** |
| --- | --- | --- | --- | --- |
| Brassicaceae | 29.573 |  | Aizoaceae | 0.159 |
| Asteraceae | 28.174 |  | orchard | 0.127 |
| Convolvulaceae | 8.632 |  | Nymphaeaceae | 0.109 |
| Musaceae | 5.659 |  | Malvaceae | 0.089 |
| Fabaceae | 4.561 |  | Passifloraceae | 0.074 |
| Solanaceae | 4.307 |  | Cactaceae | 0.072 |
| Amaryllidaceae | 4.069 |  | Agavaceae | 0.062 |
| Apiaceae | 4.045 |  | Oxalidaceae | 0.058 |
| Sapindaceae | 1.483 |  | Orchidaceae | 0.032 |
| Poaceae | 1.186 |  | Moraceae | 0.024 |
| Chenopodiaceae | 1.115 |  | Heliconiaceae | 0.015 |
| Cucurbitaceae | 1.064 |  | Lliiaceae | 0.015 |
| Amaranthaceae | 0.981 |  | Cactus | 0.012 |
| Lamiaceae | 0.596 |  | Rhamnaceae | 0.01 |
| Myrtaceae | 0.566 |  | Basellaceae | 0.008 |
| Euphorbiaceae | 0.457 |  | Asphodelaceae | 0.006 |
| Alismataceae | 0.43 |  | Plantaginaceae | 0.005 |
| Anacardiaceae | 0.417 |  | Caprifoliaceae | 0.004 |
| Dioscoreaceae | 0.406 |  | Pedaliaceae | 0.004 |
| Araceae | 0.334 |  | Acanthaceae | 0.003 |
| Caricaceae | 0.298 |  | Annonaceae | 0.003 |
| Rosaceae | 0.298 |  | Compositae | 0.003 |
| Zingiberaceae | 0.282 |  | Cyperaceae | 0.001 |
| Rutaceae | 0.17 |  |  |  |
