# Supplementary material for: Environmental factors affecting honey bees (Apis cerana) and cabbage white butterflies (Pieris rapae) at urban farmlands
Source: PeerJ. 2023 Jul 26;11:e15725. doi: 10.7717/peerj.15725 (PMC10386823; doi:10.7717/peerj.15725)
Supplement: Supplemental Information 2 — Local scale variables are crop diversity (cropdiv50), percent cover of weedy vegetation (weed50), and percent cover of the mustard family crops (Brassicaceae). Landscape scale variables include percent crop cover (crop500), percent cover of weedy vegetation (weed500), and patch density (pd1000). [file peerj-11-15725-s002.docx]

Table S2. Four generalized linear models used in the analysis of Cabbage white butterfly (*Pieris rapae*). Local scale variables are crop diversity (cropdiv50), percent cover of weedy vegetation (weed50), and percent cover of the mustard family crops (Brassicaceae). Landscape scale variables include percent crop cover (crop500), percent cover of weedy vegetation (weed500), and patch density (pd1000).

| Model | Explanatory variables |
| --- | --- |
| Null (intercept only) | – |
| Local | cropdiv50 + weed50 + Brassicaceae |
| Landscape | crop500 + weed500 + pd1000 |
| Full | cropdiv50 + weed50 + Brassicaceae + crop500 + weed500 + pd1000 |
