# Supplementary material for: Environmental factors affecting honey bees (Apis cerana) and cabbage white butterflies (Pieris rapae) at urban farmlands
Source: PeerJ. 2023 Jul 26;11:e15725. doi: 10.7717/peerj.15725 (PMC10386823; doi:10.7717/peerj.15725)
Supplement: Supplemental Information 3 — Note that in “Both” year analysis, all models include year variable. Abbreviations: flower, percent cover of flowering plants; cropdiv, crop diversity; weed, percent cover of weedy vegetation; crop, percent crop cover; pd, patch density; 5, fine scale; 50, local scale; 500 and 1,000, landscape scales. [file peerj-11-15725-s003.docx]

| Year | Model | Explanatory variables |
| --- | --- | --- |
| 2021 | Null | – |
|  | Fine | flower + cropdiv5 |
|  | Local | cropdiv50 + weed50 |
|  | Landscape | crop500 + weed500 + pd1000 |
|  | Fine+local | flower + cropdiv5 + cropdiv50 + weed50 |
|  | Fine+landscape | flower + cropdiv5 + crop500 + weed500 + pd1000 |
|  | Local+landscape | cropdiv50 + weed50 + crop500 + weed500 + pd1000 |
|  | Full | flower + cropdiv5 + cropdiv50 + weed50 + crop500 + weed500 + pd1000 |
| Both  (2020 and 2021) | Null | year |
|  | Fine | year + cropdiv5 |
|  | Local | year + cropdiv50 + weed50 |
|  | Landscape | year + crop500 + weed500 + pd1000 |
|  | Fine+local | year + cropdiv5 + cropdiv50 + weed50 |
|  | Fine+landscape | year + cropdiv5 + crop500 + weed500 + pd1000 |
|  | Local+landscape | year + cropdiv50 + weed50 + crop500 + weed500 + pd1000 |
|  | Full | year + cropdiv5 + cropdiv50 + weed50 + crop500 + weed500 + pd1000 |
