# Supplementary material for: Environmental factors affecting honey bees (Apis cerana) and cabbage white butterflies (Pieris rapae) at urban farmlands
Source: PeerJ. 2023 Jul 26;11:e15725. doi: 10.7717/peerj.15725 (PMC10386823; doi:10.7717/peerj.15725)
Supplement: Supplemental Information 4 — Abbreviation: SE, standard error; 95%LL and 95%UL, lower and upper 95% confidence intervals, respectively. Parameter: flower, percent cover of flowering plants; cropdiv, crop diversity; weed, percent cover of weedy vegetation; crop, percent crop cover; pd, patch density; 5, fine scale; 50, local scale; 500 and 1,000, landscape scales. [file peerj-11-15725-s004.docx]

| Response variable | Parameter | Estimate | SE | 95%LL | 95%UL |
| --- | --- | --- | --- | --- | --- |
| Cabbage White Butterfly  (Full model) | Intercept | 0.824 | 0.027 | 0.772 | 0.876 |
|  | weed50 | -0.021 | 0.030 | -0.080 | 0.040 |
|  | cropdiv50 | 0.153 | 0.033 | 0.086 | 0.219 |
|  | Brassicaceae | 0.035 | 0.030 | -0.025 | 0.096 |
|  | crop500 | -0.146 | 0.034 | -0.211 | -0.081 |
|  | weed500 | -0.104 | 0.029 | -0.161 | -0.047 |
|  | pd1000 | 0.002 | 0.030 | -0.056 | 0.062 |
|  | | | | | |
| Asian honey bee  – 2021 | Intercept | 0.476 | 0.099 | 0.279 | 0.673 |
|  | flower | 0.355 | 0.079 | 0.198 | 0.513 |
|  | cropdiv5 | 0.022 | 0.109 | -0.194 | 0.238 |
|  | cropdiv50 | -0.111 | 0.110 | -0.330 | 0.107 |
|  | weed50 | 0.218 | 0.090 | 0.041 | 0.396 |
|  | crop500 | 0.108 | 0.104 | -0.099 | 0.315 |
|  | weed500 | -0.238 | 0.101 | -0.438 | -0.038 |
|  | pd1000 | -0.136 | 0.102 | -0.338 | 0.066 |
| Asian honey bee  – Both  (2020 and 2021) | Intercept | 0.038 | 0.168 | -0.294 | 0.369 |
|  | year | 0.478 | 0.228 | 0.028 | 0.928 |
|  | cropdiv5 | 0.129 | 0.128 | -0.123 | 0.381 |
|  | cropdiv50 | -0.065 | 0.103 | -0.268 | 0.139 |
|  | weed50 | 0.247 | 0.084 | 0.08 | 0.413 |
|  | crop500 | 0.268 | 0.108 | 0.055 | 0.48 |
|  | weed500 | -0.336 | 0.102 | -0.537 | -0.134 |
|  | pd1000 | -0.235 | 0.103 | -0.438 | -0.031 |
